# A retrospective controlled study comparing Spinecor vs exercises for Adolescent Idiopathic Scoliosis

**DOI:** 10.1186/1748-7161-8-S2-O57

**Published:** 2013-09-18

**Authors:** Fabio Zaina, Fabio Digiacomo, Fabio Zaina, Michele Romano, Alessandra Negrini, Sabrina Donzelli, Monia Lusini, Salvatore Minnella, Stefano Negrini

**Affiliations:** 1 ISICO Italian Scientific Spine Institute, Milan, Italy

## Background

SpineCor and exercises both have results testifying to their effectiveness in Adolescent Idiopathic Scoliosis (AIS) treatment. Several years ago, we introduced SpineCor as a treatment for patients at the highest risk of bracing which we previously treated with exercises. In a previous study, we compared the short-time results of SpineCor and specific exercise for AIS. 

## Purpose

The objective of this study was to compare the end treatment results of the SpineCor vs. Scientific Exercises Approach to Scoliosis (SEAS) for AIS. 

## Methods

Study design: retrospective controlled study. Population: Exercise Group (EG): 28 consecutive scoliosis patients (26 females), age 13±2, TRACE 4.5, Cobb angle 18±3°, ATR 7±3°, Risset 0-3. 

SpineCor Group (SG): 41 patients (33 females), age 13±1, TRACE score 6, Cobb angle 24±5°; ATR 8±3°, Risser 0-3. EG patients performed specific exercises twice per week according to the SEAS protocol. SG patients wore the SpineCor brace 20 hours per day. Patients were evaluated both clinically and radiographically before and after the treatment. Main outcome measured TRACE (changes ≥3), Cobb angle (changes > ±5) and ATR. 

## Results

At baseline, the Cobb angle in the SG was significantly larger than the EG group. Considering the number of patients with significant changes for Cobb angle, we found 7% improved, 36% stable and 57% worsened vs. 22%, 32% and 46% respectively (p>0.05). For ATR, the results were similar. There were similar results for TRACE: 46% improved and 54% stable for EG versus 65%, 31% and 4% worsened in the SG (P>0.05). 

## Conclusions and discussion

There was a slight difference among groups at the beginning of the study, so the interpretation of the results must be cautious. Both treatments could achieve some improvements of the aesthetics. In terms of risk of progression, results were slightly better for SpineCor, but the difference is not statistically significant. This could be due to the difference in the initial Cobb angle: the SpineCor group included more severe curves compared to the exercise group. The main limits of the study were the retrospective design and the small population. Moreover, the initial difference in the severity of the curve made the comparison not totally reliable. 
